# Vitamin D Deficiency Is Associated with the Presence and Severity of Diabetic Retinopathy in Type 2 Diabetes Mellitus

**DOI:** 10.1155/2015/374178

**Published:** 2015-05-20

**Authors:** Nuria Alcubierre, Joan Valls, Esther Rubinat, Gonzalo Cao, Aureli Esquerda, Alicia Traveset, Minerva Granado-Casas, Carmen Jurjo, Didac Mauricio

**Affiliations:** ^1^Biomedical Research Institute of Lleida, University of Lleida, Rovira Roure 80, 25198 Lleida, Spain; ^2^Biostatistics & Epidemiology Unit, Biomedical Research Institute of Lleida, University of Lleida, Rovira Roure 80, 25198 Lleida, Spain; ^3^Department of Endocrinology & Nutrition, University Hospital Arnau de Vilanova, Rovira Roure 80, 25198 Lleida, Spain; ^4^Department of Laboratory Medicine, University Hospital Arnau de Vilanova, Rovira Roure 80, 25198 Lleida, Spain; ^5^Department of Ophthalmology, University Hospital Arnau de Vilanova, Rovira Roure 80, 25198 Lleida, Spain; ^6^Department of Endocrinology & Nutrition, Health Sciences Research Institute and University Hospital Germans Trias i Pujol, Carretera Canyet S/N, 08916 Badalona, Spain

## Abstract

There is very few evidences on the role of vitamin D in the development of diabetic retinopathy. The aim of the current study was to explore whether there is an association of vitamin D status and diabetic retinopathy in type 2 diabetes. Two groups of patients were selected: 139 and 144 patients with and without retinopathy, respectively, as assessed by an experienced ophthalmologist. Subjects with advanced late diabetic complications were excluded to avoid confounding biases. 25-Hydroxy-vitamin D_3_ (25(OH)D) concentrations and vitamin D deficiency were associated with the presence of diabetic retinopathy. Additionally, patients with more advanced stages of retinopathy (grades 2–4) had lower concentrations of 25(OH)D and were more frequently vitamin D deficient as compared with patients not carrying this eye complication. In conclusion, our study confirms the association of vitamin D deficiency with the presence and severity of diabetic retinopathy in type 2 diabetes. Further experimental and prospective studies on this issue are clearly warranted.

## 1. Introduction

Vitamin D deficiency is highly prevalent worldwide [1]. Serum 25-hydroxy-vitamin D_3_ (25(OH)D) is a better indicator of vitamin D sufficiency than the active hormone, that is, 1,25-dihydroxy-vitamin D_3_ [2]. Therefore, the serum concentration of 25(OH)D is widely accepted as a good indicator of the status of vitamin D in a given subject.

The main biological actions of vitamin D include the maintenance of mineral homeostasis and the regulation of bone remodeling [1]. However, there is a vast array of pleiotropic actions of this vitamin that were already recognized more than two decades ago [2]. This area of investigation led to improved knowledge on the potential role of vitamin D on glucose homeostasis and in the pathogenesis of type 2 diabetes. Multiple studies have previously shown that vitamin D deficiency is highly prevalent in type 1 and type 2 diabetes [3]. Additionally, there is a growing interest on the potential role of vitamin in the development of diabetic micro- and macroangiopathic complications [4, 5].

Further, the potential role of vitamin D in the development of diabetic retinopathy has been a matter of specific interest in recent years. There is some experimental evidence on the preventive effect of vitamin D in the development of diabetic retinopathy in a rodent model [6]. However, the evidence behind the involvement of vitamin D is really scarce. A recent PubMed search (accessed on April 24, 2015), with the use of terms “vitamin D” and “diabetic retinopathy,” yielded only 31 publications. Thus, other studies have already addressed the question of the potential implication of vitamin D in the pathogenesis of diabetic retinopathy [7–15]. Among these studies there were only 7 publications that included the study of the potential association between vitamin D and the presence of this microangiopathic complication in patients with type 2 diabetes [7–13]. Because of the cross-sectional design of these studies, only an association between lower serum 25(OH)D concentrations and/or vitamin D deficiency and the presence of diabetic retinopathy in type 2 diabetes has been described. Additionally, there are also conflicting data on the role of vitamin D in retinopathy in type 1 diabetes [4, 14, 15]. In a cross-sectional study, Shimo et al. showed an association between vitamin D deficiency and the presence of diabetic retinopathy in a small sample of young adults with type 1 diabetes [15]. However, the only prospective study in this field was performed also in type 1 diabetes and did not confirm vitamin D deficiency as a risk factor for diabetic retinopathy or any other microvascular complications [14].

To our knowledge, there are no data on the possible association of vitamin D and diabetic retinopathy in type 2 diabetic patients in any European population, including Spain. Thus, the primary objective of the current study was to assess whether there is an association of vitamin D status and diabetic retinopathy in type 2 diabetes. Additionally, as a secondary outcome, we aimed to assess the association of vitamin D concentrations and the frequency of its deficiency with the severity of retinopathy.

## 2. Patients, Material, and Methods

### 2.1. Participants

This was an observational case-control study. Study subjects were selected from a group of participants in a study on differential characteristics of type 2 diabetic patients with (cases) and without retinopathy (controls) in our center. The study was done at the University Hospital Arnau de Vilanova in Lleida, Spain. This is a tertiary referral center for ophthalmology and endocrinology in the region. A detailed description of the study population has been recently published [16]. From a total number of 299 subjects, we could assess a total of 283 subjects with complete data for the current main study outcomes, as explained below. We took advantage of using the previous study design to assess the association of diabetic retinopathy and vitamin D status in subjects without other advanced diabetic late complications. All study subjects had normal kidney function (calculated glomerular filtration rate > 60 mL/min). By study design, all subjects without retinopathy had normal albumin-to-creatinine ratio (<30 mg/g) and those with retinopathy had no macroalbuminuria (albumin-to-creatinine ratio < 300 mg/g). All study subjects had no known cardiovascular complications (coronary disease, cerebrovascular disease, or peripheral artery disease). Also, none of the patients had a history of diabetic foot disease. We took advantage from the design and performance of a study on quality of life in patients with diabetic retinopathy [16]. In that study, we included a total number of 299 type 2 diabetic subjects. From the original study population, 16 patients could not be included because of the following reasons: 2 patients did not accept biobanking of blood samples at recruitment, serum samples were not available from 8 additional subjects, and 6 were not included because they were taking oral supplements containing vitamin D and/or calcium. Thus, a final number of 139 patients with retinopathy and 144 without this complication were included. All subjects underwent a complete clinical and laboratory evaluation.

### 2.2. Methods

Demographic and clinical data relevant to the proper conduct of the present study were recorded and are shown in Table 1. Blood pressure was measured in the sitting position after resting for 10 minutes using a blood pressure monitor (HEM-7001E, Omron, Spain). Hypertension or dyslipidemia were considered present when the patient was being treated with antihypertensive or lipid-lowering drugs, respectively. Weight and height were measured by standardized methods, and the body mass index was then calculated (expressed as kg/m^2^).

Blood and spot urine samples were obtained from all participants in the fasting state. Standard laboratory procedures were used to determine the basic blood and urine biochemistry variables on a Hitachi Modular DDPP analyzer (Roche Diagnostics, Indianapolis, USA). Glycated hemoglobin was measured with a HPLC analyzer Variant II Turbo (Bio-RAD, Hercules, USA). Intact parathormone was determined by electrochemiluminescence immunoassay in an Elecsys E170 analyzer (Roche Diagnostics, Indianapolis, USA), with an intra- and interassay variability of 2.2% and 6%, respectively. Serum concentrations of 25(OH)D were measured by a chemiluminescent microparticle immunoassay in an Architect i2000SR analyzer (Abbott Diagnostics, Lake Forest, USA), with an intra- and interassay variability of 2.3% and 6.2%, respectively. The date of blood testing was used to classify the season of assessment, as follows: winter (January–March), spring (April–June), summer (July–September), and autumn (October–December).

An experienced ophthalmologist explored the retina and classified the retinopathy status according to an internationally accepted classification [17]. To assess physical activity, we used the concept of active leisure time, which defines a sedentary person as one who spends less than 10% of his/her daily energy expenditure performing any physical activity that requires at least 4 METs (equal or greater physical activity expenditure than brisk walking for 30 minutes) [18]; this allowed us to classify each subject as sedentary or active. Dietary intake of vitamin D and calcium was measured by a validated 101-food-item semiquantitative food frequency questionnaire (available at http://bibliodieta.umh.es/files/2011/07/CFA101.pdf) [19]. Nutrient values for vitamin D and calcium were obtained from food composition tables of the US Department of Agriculture and supplemented with Spanish sources [19, 20].

The study protocol was approved by the local ethics committee, in accordance with the Declaration of Helsinki. All participants signed the written informed consent form.

As this is an ancillary study of our previous one, in which only patients with relevant data were included, before the analysis we assumed a difference of at least 3 ng/mL in 25(OH)D concentrations between patients with and without retinopathy with a standard deviation of 8 units. The inclusion of 139 with and 144 patients without retinopathy allowed the achievement of a statistical power of 93% (beta = 0.07) to detect such difference, using a one-sided* t*-test to assess the differences with 5% of significance (alpha = 0.05). Therefore, we were able to address the analysis of the primary outcome with sufficient post hoc statistical power.

### 2.3. Statistical Analysis

Mean (and standard deviations) or absolute and relative frequencies (in percentages) were computed for quantitative or qualitative variables, respectively. Differences between groups were assessed by means of Mann-Whitney tests or Chi-squared tests as adequate. Vitamin D deficiency was defined for different thresholds (at each unit from 15 to 40 pg/mL) of the serum concentrations of 25(OH)D, and the relative risk was computed to assess the increased risk of vitamin D deficiency in patients with retinopathy versus those without this complication. To further analyze the association of retinopathy with vitamin D, linear regression models were used to assess serum concentrations of 25(OH)D and logistic regression models to assess vitamin D deficiency, establishing an appropriate threshold in the serum levels for this purpose. Multivariate regression analyses were also performed including age, sex, race, hemoglobin A_1c_, creatinine, disease duration, body mass index, hypolipidemic treatment, and physical activity as potential predictors in addition to patient group. The final models were obtained by applying a stepwise algorithm to minimize Akaike's Information Criterion. The interaction of the patient group (DR and non-DR) with the creatinine serum was included in the models as it was shown to be a potential and significant predictor. A significance level of 0.05 was used. Statistical analyses were conducted with R 3.0.1 (R foundation for Statistical Computing, Vienna, Austria: http://www.r-project.org/).

## 3. Results

**Figure 1:**
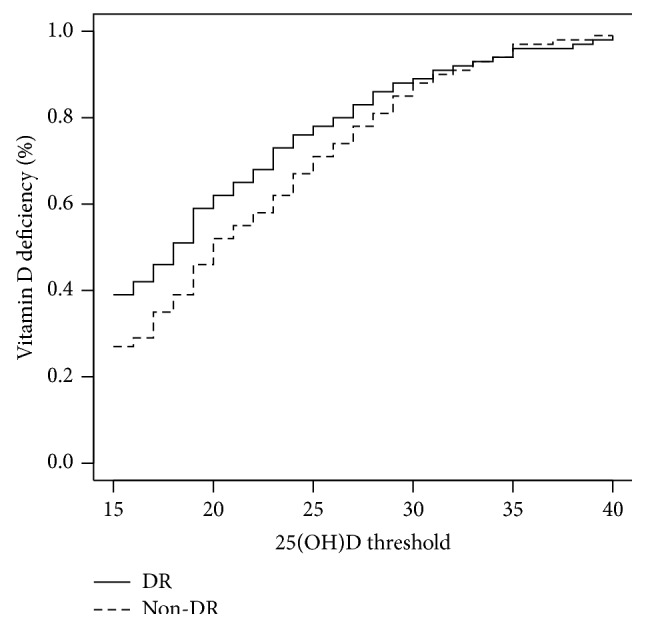
Frequency of vitamin D deficiency according to different 25(OH)D thresholds for patients with and without retinopathy. 25(OH)D: 25-hydroxy-vitamin D_3_; DR: diabetic retinopathy.

Table 1 shows the results of the different study variables and the differences between patients with and without retinopathy. As previous studies that compared subjects with and without this diabetic late complication, those with retinopathy had longer diabetes duration, had higher glycated hemoglobin concentrations, and were more frequently on antihypertensive and insulin treatments. Interestingly, the dietary intake of vitamin D and calcium was very similar in both groups of patients. No differences were observed in other clinical and laboratory parameters in the unadjusted analysis of the data. Although 25(OH)D concentrations were lower in patients with retinopathy, this difference reached only borderline statistical difference (*p* = 0.05). Additionally, the frequency of vitamin D deficiency, defined as serum 25(OH)D below 20 ng/mL, was higher in subjects with retinopathy, but this difference did not reach statistical significance (*p* = 0.07). Nevertheless, the prevalence of retinopathy for 25(OH)D thresholds below 20 mg/mL was significantly higher (thresholds 15 to 19; *p* ≤ 0.05), and the associated relative risk of retinopathy was higher for all these thresholds (Figure 1). For the purpose of the multivariate analysis, a concentration of 15 ng/mL was chosen as the threshold to define vitamin deficiency; in the previous analysis, this threshold yielded the highest relative risk of retinopathy (RR: 1.43; *p* = 0.03).

The multivariate analyses showed that there was a significant association of retinopathy and 25(OH)D, even when considering other variables associated with this variable. These results were consistent when analyzing both the concentrations of 25(OH)D and the presence of vitamin D deficiency, as defined by a 25(OH)D <15 ng/mL (*p* values 0.04 and 0.009, resp.) (Table 2). In addition to this, Caucasian patients and physically active patients had significantly higher 25(OH)D levels (*p* values 0.05 and 0.03, resp.) (Table 2) and significantly lower vitamin D deficiency risk (*p* values 0.009 and 0.004, resp.). Body mass index was inversely associated with 25(OH)D concentrations and positively associated with vitamin D deficiency (*p* values 0.009 and 0.004, resp.) (Table 2). Considering winter as the reference season associated with lower vitamin D levels (one as risk value), the other seasonal 25(OH)D concentrations during autumn, spring, and summer were statistically higher in the two models (Table 2). Interestingly, creatinine concentration interacted with the patient group (retinopathy versus no retinopathy) in both models (*p* values 0.03 and 0.003). To further illustrate this interaction (Table 3), the stratification of creatinine concentrations by tertiles in each of the 2 study groups revealed that patients with retinopathy had frequencies of vitamin D deficiency that augmented with increasing creatinine concentration tertiles (31.9%, 34.8%, and 46.7%, resp.) in contrast to the frequencies observed in the group of patients without retinopathy.

Further to the main analysis, we explored the association of vitamin D status with the severity of retinopathy. The distribution of subjects with retinopathy according to its severity was as follows: grade 1: 63; grade 2: 42; grade 3: 28, and grade 4: 6. We analyzed the differences among patients without retinopathy and those with this complication stratified according to its severity (grade 1 versus grades 2–4). The results of this analysis are shown in Table 4. Interestingly, patients with advanced retinopathy (grades 2–4) were more frequently vitamin D deficient (25(OH)D <20 ng/mL) and had lower mean concentration of 25(OH)D compared with type 2 diabetic patients without retinopathy (*p* values of 0.03 and 0.02, resp.) (Table 4). Concerning these variables, patients with mild diabetic retinopathy (grade 1) were not significantly different to the group without retinopathy.

## 4. Discussion

Our study confirms the association of vitamin D deficiency with diabetic retinopathy in type 2 diabetes. As in previous reports, type 2 diabetic patients with retinopathy had lower serum 25(OH)D concentrations. Furthermore, a higher proportion of vitamin D deficiency was confirmed in subjects carrying this microvascular complication. In addition to this, there was an increased risk of retinopathy in those with severe vitamin D deficiency (RR = 1.43, *p* = 0.03), with this association being even more significant in the adjusted model that included the interaction of retinopathy with creatinine (*p* = 0.009). Concerning the severity of diabetic retinopathy, patients with more advanced stages of retinopathy had lower concentrations of 25(OH)D and were more frequently vitamin D deficient.

This is the first study to address the potential relationship of vitamin D status with diabetic retinopathy in type 2 diabetes in Europe. Previous publications analyzed the data according to differences in 25(OH)D concentrations between groups or, alternatively, as the prevalence of retinopathy according to a given 25(OH)D threshold to define deficiency of this vitamin. An initial study on 66 Turkish type 2 diabetic subjects (46 with retinopathy) found no differences in serum 25(OH)D between type 2 diabetic patients according to the presence or absence of retinopathy and also to the severity of retinopathy [7]. In contrast, Suzuki et al. showed that Japanese type 2 diabetic patients with proliferative retinopathy had lower serum 25(OH)D [8]. Also, the first study carried out in the USA revealed an association of 25(OH)D concentrations with the severity of diabetic retinopathy [9]. However, the only population-based study on this matter, performed also in the USA, could not demonstrate an association between retinopathy and serum 25(OH)D concentration; these authors only found a greater prevalence of vitamin D deficiency with increased severity of retinopathy [10]. In Lebanese type 2 diabetic patients with retinopathy, lower concentrations of 25(OH)D were also found; additionally, vitamin D was an independent predictor of retinopathy [11]. A very recent Chinese study, with the largest number of patients with diabetic retinopathy included in a single study so far, revealed an independent association between 25(OH)D concentrations and diabetic retinopathy, especially for advanced sight-threatening retinopathy [12]. This study found a twofold increase in sight-threatening retinopathy among subjects with serum 25(OH)D below 15.57 ng/mL. A recent large, population-based, cross-sectional study in Korea confirmed that there is an inverse relationship of 25(OH)D concentrations with the presence of any retinopathy and also with proliferative retinopathy [13]. This study did not provide details on the type of diabetes of the included subjects. Thus, most of the reports were able to identify an association between 25(OH)D and retinopathy or its deficiency. Additionally, those studies that performed a detailed characterization of the severity of retinopathy revealed that proliferative or advanced retinopathy grades were clearly associated with 25(OH)D and/or its deficiency [9, 10, 12, 13]. It should be noted that the few available studies on this issue in type 1 diabetes show conflicting data [14, 15]. In summary, our results are discordant with 2 previous studies that did not show an association of vitamin D with diabetic retinopathy in type 2 diabetes [7, 10]. In contrast, our findings are in line with the majority of previous reports that demonstrated that vitamin D status is associated with the presence of diabetic retinopathy [9, 11–13]. Additionally, it is remarkable that, as in our study, most others have found an association of vitamin D status and the severity of retinopathy [9–13].

Our study shares the most important limitation with previous studies; that is, all of them were cross-sectional. This design allows only for the identification of an association between study variables. To our knowledge, there is no prospective follow-up study in type 2 diabetes and the only one available in type 1 diabetes did not identify vitamin D as a factor involved in the development of retinopathy [4]. Thus, the question on the potential role of vitamin D in the pathogenesis of diabetic retinopathy has not been answered yet. Another limitation resides on the fact that only one of the previous studies can be considered as population-based; thus, the external validity of the findings of all other reports, including ours, is clearly limited. Although subjects included in our study may not be fully representative of the background population, the clinical characteristics of patients in our study are very similar to those of recently published population-based cohorts in our region [21].

Another limitation of our study is that we did not determine the time of sun exposure. This limitation is also shared with most previous studies, except for the one by Ahmadieh et al. who used a questionnaire to assess the time spent outdoors [11]. Physical activity may be used as a surrogate of sun exposure as most of this activity is performed outdoors. Thus, we used this measure as an additional variable in the multivariate analysis that revealed that this was a variable with an influence on vitamin D status. Therefore, future studies should include a measure of sun exposure as an essential contributor to 25(OH)D concentrations.

We should point out that none of the subjects in the current study had advanced renal disease and/or impairment. It must be pointed out that renal disease in diabetes could contribute as an important confounder of vitamin D status [1, 22]. Some of the previous studies either did not provide information on renal function or included patients with advanced renal disease. Our findings concerning the strong interaction of serum creatinine and the retinopathy status should be taken into consideration in future studies. The careful revision of the previous publications on this matter confirmed that only the American population-based study by Patrick et al. included creatinine as a potential important confounder in the analysis of the association of 25(OH)D and retinopathy.

Besides, an association between vitamin D and cardiovascular disease has been previously described [21], also in type 2 diabetes [23]. The presence of macrovascular disease in patients with type 2 diabetes may be a confounding factor when assessing an association of vitamin D with other conditions. In our study, the presence of clinical cardiovascular disease was an exclusion criterion. However, the potential effect of the presence of atherosclerotic disease was not controlled for in any of the previous studies. Four of them did not provide information on the presence of cardiovascular disease [7, 8, 10, 11], and the other 2 included a significant proportion of patients with cardiovascular disease [9, 12]. The exclusion of this complication in our study clearly avoided the potential association of vitamin D with cardiovascular disease as a confounding variable. In conclusion, an important strength of the current study is that the patients included did not have important confounding conditions that have an important influence on vitamin D concentrations, that is, renal insufficiency and cardiovascular disease.

## 5. Conclusion

Our study confirms the association of a higher frequency of vitamin D deficiency and lower concentrations of 25(OH)D with diabetic retinopathy in patients with type 2 diabetes. Further, these parameters of poor vitamin D status are also associated with the severity of diabetic retinopathy. These findings reveal the potential role of vitamin D in the pathogenesis of diabetic retinopathy. However, we are in great need of well-designed prospective observational studies sufficiently powered to test the role of vitamin D status in the development of diabetic retinopathy and other diabetic microvascular complications.

## Figures and Tables

**Table 1:** Clinical and biochemical characteristics of patients with type 2 diabetes with and without retinopathy.

	No retinopathy (*n* = 144)	Retinopathy (*n* = 139)	*p* value
Sex (male/female)	74/70	71/68	1
Non-Caucasian	5 (3.5%)	6 (4.3%)	0.95
Age (yr)	58.1 (10.3)	60.3 (8.9)	0.09
Disease duration (yr)	7.2 (5.5)	13.9 (9.3)	<0.01
Insulin treatment (with or without oral agents)	17	74	<0.01
Smoking (current/past/never)	30/49/64	28/41/70	0.61
Hypolipidemic treatment	65 (45.1%)	66 (47.5%)	0.78
Antihypertensive treatment	68 (47.2%)	94 (67.6%)	<0.01
Body mass index (kg/m^2^)	31.2 (5.2)	31.8 (5.4)	0.32
Hemoglobin A_1c_ (%)	7.3 (1.2)	8.3 (1.4)	<0.01
Cholesterol (mg/dL)	186.1 (37)	185.1 (36.5)	0.82
HDL cholesterol (mg/dL)	48.6 (10.9)	52.2 (15.6)	0.08
LDL cholesterol (mg/dL)	111.6 (30.9)	106.4 (30.2)	0.19
Triglycerides (mg/dL)	137.4 (82.4)	140.5 (122.7)	0.64
Serum creatinine (mg/dL)	0.8 (0.2)	0.8 (0.2)	0.83
Urinary albumin-to-creatinine ratio, (mg/g)	11 (26.2)	34.8 (52.4)	<0.01
Calcium (mg/dL)	9.3 (0.4)	9.3 (0.4)	0.69
Phosphate (mg/dL)	3.5 (0.5)	3.5 (0.4)	0.38
Parathormone (pg/mL)	48.1 (18.9)	45.3 (17.0)	0.11
25(OH)D (ng/mL)	20.5 (8.1)	19.2 (10.1)	0.05
Vitamin D deficiency (25(OH)D <20 ng/mL)	73 (50.7%)	86 (61.9%)	0.07
Season of assessment (spring/summer/autumn/winter)	38/55/18/33	37/42/26/34	0.37
Physical activity (active/sedentary)	74/70	74/65	0.85
Daily dietary vitamin D intake (mg)	4.4 (2.2)	4.6 (2.5)	0.93
Daily dietary calcium intake (mg)	1176 (434)	1153 (529)	0.65

Data are mean ± standard deviation or n (%), as needed. HDL: high density lipoprotein; LDL: low density lipoprotein; 25(OH)D: 25-hydroxy-vitamin D_3_.

**Table 2:** Multivariate analysis for the association of the status of retinopathy and 25(OH)D. The relevant parameters and *p* values from the linear and logistic regression models to evaluate the concentrations of 25(OH)D and the presence of vitamin D deficiency (defined as 25(OH)D <15 ng/mL) are shown. For qualitative variables, the category in the model is provided. The reference categories are no retinopathy, Caucasian, Winter and sedentary for patient group, race, season, and physical activity, respectively.

	Linear model 25(OH)D concentration		Logistic model deficiency (25(OH)D <15 ng/mL)	
	Beta (SE)	*p* value	Beta (SE)	*p* value
Intercept	20.52 (5.17)		−0.73 (1.49)	
Patient group (retinopathy)	10.36 (4.98)	0.04	−3.76 (1.44)	0.009
Creatinine	2.53 (4.25)	0.55	−1.76 (1.33)	0.19
Ethnicity (non-Caucasian)	−5.07 (2.67)	0.05	1.94 (0.74)	0.009
Body mass index	−0.26 (0.10)	0.009	0.08 (0.03)	0.004
Season (autumn)	3.21 (1.68)	0.05	−0.74 (0.42)	0.08
Season (spring)	5.03 (1.64)	0.002	−1.03 (0.42)	0.02
Season (summer)	8.04 (1.59)	<0.00001	−2.09 (0.46)	<0.00001
Physical activity (active)	2.21 (1.04)	0.03	−0.87 (0.30)	0.004
Interaction group *∗* creatinine	−13.32 (6.03)	0.03	5.17 (1.77)	0.003

25(OH)D: 25-hydroxy-vitamin D_3_; SE: standard error.

**Table 3:** Frequency of vitamin D deficiency, defined as 25(OH)D <15 ng/mL, according to tertiles of serum creatinine concentrations in the 2 study groups.

Study group	Serum creatinine concentration (mg/dL)		
	Tertile 1 (<0.72)	Tertile 2 (0.72–0.88)	Tertile 3 (>0.88)
No retinopathy	17/47 (36.2%)	12/51 (23.5%)	10/46 (21.7%)
Retinopathy	15/47 (31.9%)	16/46 (34.8%)	21/45 (46.7%)

Values are *n* (%).

**Table 4:** Results of the analysis of serum 25(OH)D concentrations and frequency of vitamin D deficiency according to the severity of diabetic retinopathy.

Vitamin D status	No DR	DR: grade 1	DR: grades 2–4	*p* values	
	*n* = 144	*n* = 63	*n* = 76	Grade 1 versus no DR	Grades 2–4 versus no DR
25 (OH)D (ng/mL)	20.5 (8.1)	20 (9)	18.6 (11)	0.52	0.02
Vitamin D deficiency (<20 ng/dL)	73 (50.7%)	35 (55.6%)	51 (67.1%)	0.62	0.03

Data are mean ± standard deviation or *n* (%), as needed. DR: diabetic retinopathy; 25(OH)D: 25-hydroxy-vitamin D_3_.
